# Relating trophic resources to community structure: a predictive index of food availability

**DOI:** 10.1098/rsos.160515

**Published:** 2017-02-08

**Authors:** Valerio Zupo, Timothy J. Alexander, Graham J. Edgar

**Affiliations:** 1 Stazione Zoologica Anton Dohrn, Integrative Marine Ecology Department, Benthic Ecology Center, Punta San Pietro, Ischia80077, Italy; 2 Department of Fish Ecology and Evolution, Centre of Ecology, Evolution and Biogeochemistry, EAWAG Swiss Federal Institute of Aquatic Science and Technology, Seestrasse 79, Kastanienbaum 6047, Switzerland; 3 Division of Aquatic Ecology and Evolution, Institute of Ecology and Evolution, University of Bern, Baltzerstrasse 6, Bern 3012, Switzerland; 4 Institute for Marine and Antarctic Studies, University of Tasmania, GPO Box 252-49, Hobart, Tasmania 7001, Australia

**Keywords:** food webs, trophic groups, feeding guilds, abundance, resources

## Abstract

The abundance and the distribution of trophic resources available for consumers influence the productivity and the diversity of natural communities. Nevertheless, assessment of the actual abundance of food items available for individual trophic groups has been constrained by differences in methods and metrics used by various authors. Here we develop an index of food abundance, the framework of which can be adapted for different ecosystems. The relative available food index (RAFI) is computed by considering standard resource conditions of a habitat and the influence of various generalized anthropogenic and natural factors. RAFI was developed using published literature on food abundance and validated by comparison of predictions versus observed trophic resources across various marine sites. RAFI tables here proposed can be applied to a range of marine ecosystems for predictions of the potential abundance of food available for each trophic group, hence permitting exploration of ecological theories by focusing on the deviation from the observed to the expected.

## 1. Introduction

### 1.1. The importance of trophic resources

Nutrient supply and productivity gradients can strongly influence the diversity of natural communities through trophic linkages [1,2]. Consequently, attempts to predict biodiversity patterns in marine ecosystems should consider the abundance of food available for different trophic groups [3,4]. To date, research has been focused primarily on influences of predators on prey populations, through a top-down approach [5]. Various studies also suggest that resources and consumers interact to structure food webs [6,7] with, for example, demonstration that herbivore and predator abundances vary predictably along natural productivity gradients [1].

Unfortunately, the various forms of trophic data reported among studies impede broad-scale comparisons because of different sampling methods, different trophic groups, incomplete sets of plant and animal taxa, and different units of measurements [8,9]. In the marine context, benthic and planktonic morphofunctional groups are often sampled with different instruments, on different surface areas or volumes, and among different habitats. For this reason, only a few broad-scale cross-ecosystem comparisons have yet been made on relationships between productivity/functioning and food resources available for each trophic group [3,5].

### 1.2. Prediction of trophic resources

Nevertheless, a classification of ecosystems based on the abundance of each trophic resource is theoretically possible [10]. For example, the amount of plant biomass potentially available for macroherbivores will inevitably be much higher in seagrass meadows than unvegetated sandy substrata or marine caves [11]. In addition, the abundance of food available for macrocarnivores is higher on coral reefs than shallow seaweed meadows [12,13]. Extending such generalizations, food resources available to different trophic groups can be evaluated by considering habitat constraints.

Various pressures acting locally also influence and modulate these general trends. For example, the abundance of plant detritus is high in seagrass meadows, but the presence of strong currents may disperse the detritus particles and make that resource less abundant [14]. Wave exposure and associated surge also negatively influence detritus, potentially reducing availability for herbivore–detritivores. Additionally, food for microherbivores is abundant in shallow rocky bottoms and increases with increasing nutrients [15], but declines in deep rocky environments, owing to the limiting influence of light [16]. Therefore, nutrient availability and depth are important moderating factors, with consistent effects across a range of ecosystems [17].

Our study aims at describing general patterns of relative abundance of food available for trophic groups among various marine habitats. Based on these patterns, we developed a mechanistic model of food availability and validated its predictions through comparisons of computed versus observed food resources at several comprehensively sampled sites. Trophic resources were assessed solely on the basis of their physical presence in each habitat, irrespective of whether the food material was protected by physical, chemical or behavioural defences [18]. The model is presented here in order to easily incorporate an estimate of trophic resources in evaluations of diversity–productivity relationships [19] and in other analyses of marine ecosystems.

## 2. Material and methods

### 2.1. Computation of relative available food index tables

The relative available food index (RAFI) was computed by screening the global literature on trophic resources in marine habitats (electronic supplementary material, table S1). A literature search was conducted using ISI Web of Science™ (www.webofknowledge.com) from 1945 to 2010, plus hardcopy literature contained in the library of Stazione Zoologica, Naples, that encompasses magazine collections from 1872 to the present. Studies involving abundance and taxonomic composition of marine organisms were considered when the information contained was comparable and appropriate, in terms of surface units, abundance units, substrata and taxonomic groups investigated. Restrictions related to language, publication date or publication status were not imposed. The data recorded show regional patchiness, owing to the availability of specific studies according to the distribution patterns of authors (table 1). The first step was the evaluation of the food resources available at each of five substrata (hard, soft, hard biogenic, macroalgae and seagrass beds; table 2) and for 11 trophic groups (table 3) that were expressed according to the type and size of prey items [20].

**Table 1.** Geographical distribution of the studies used for the construction of the RAFI model. The number of publications considered for each region is reported in columns, according to various ecosystems (resulting from the classification in electronic supplementary material, table S1), in rows. The total per cent contribution of researches performed in each region is reported in the last row.

	geographical areas							
biotopes	Mediterranean	Atlantic	Australian Pacific	Pacific Ocean	Indian Ocean	Caribbean Sea	China Sea	Baltic
marine caves	1	2				1		
biogenic		3	2	3	1			
hard bottoms	4	2	1	1		1	1	1
macroalgae	2	2	2	2			2	
seagrasses	15	7	3	1	3	2	1	1
soft bottoms	1	3		1	1	1		
harbours	14	3	1		1	1		1
biotope typologies	4	2	2	1	2	2		
per cent contribution	38.0	22.2	10.2	8.3	7.4	7.4	3.7	2.8

**Table 2.** Example of the ranking process applied to herbivore (He) food resources for five substrata (in rows). A score from 1 to 3 is attributed (third column) according to the ranges of abundance reported (second column). The literature used to obtain abundance ranges is indicated in the fourth column (numbers in brackets are referred to electronic supplementary material, table S1).

basic substrata	abundance of food for He	rank (1–3)	literature
soft substratum	0.3–6 g C m^−2^	1	[78,82]
hard substratum	56–234 g C m^−2^	2	[26,30,34]
hard biogenic	41–140 g C m^−2^	2	[21,22]
macroalgae beds	40–310 g C m^−2^	3	[35,38,42]
seagrass beds	20–600 g C m^−2^	3	[47,54,75,76]

**Figure 1.**
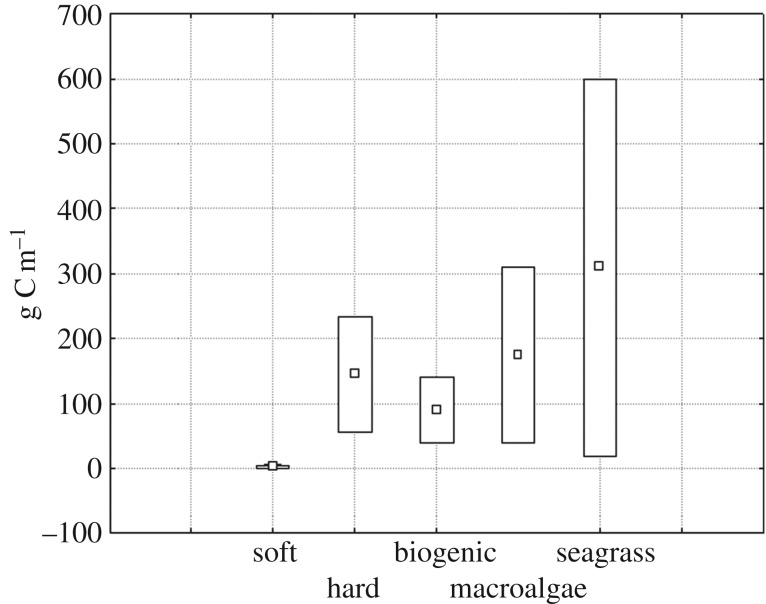
Abundances of trophic resources, expressed as g C m^−1^, available for herbivore consumers in five different substrata. The whole range (0–600 g C m^−1^) has been divided into three categories of abundance.

To calculate the abundance of food potentially available for microcarnivore (mCa) species in each substratum, for example, research articles containing data on the abundance of microcarnivore prey (meiofauna and other small animals less than 1 mm in size) were selected, and the abundances reported by different authors (in various sites, seasons, etc.) were recorded. Similarly, to evaluate the abundance of food available for macroherbivores (He) in various substrata, papers containing information on the standing crops of plants and algae were selected for each of five habitats, and abundance data were recorded (table 2, second column). Available data may be expressed in several different units (e.g. number of individuals, mg of biomass, µg of carbon or kcal per unit surface area) according to the methods followed by each author. In these cases, all data were converted, according to [21], to g C m^−2^, in order to permit comparisons among the different studies. Finally, the range of abundances recorded (figure 1) was divided into three intervals ranked 1 (low abundance), 2 (medium) and 3 (high), as indicated in table 2 (third column). The interval subdivision was made according to a best professional judgement in order to highlight the differences found among ranges.

**Figure 2.**
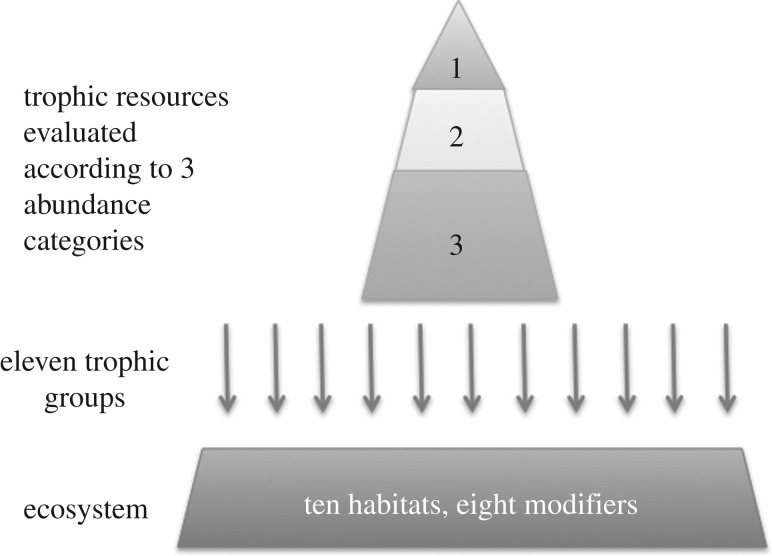
Each ecosystem is classified according to 10 broad habitats and defined according to eight specific modifiers. The trophic resources available for 11 trophic groups of consumers are evaluated according to three levels of abundance (1, low; 2, medium; 3, high).

Subsequently, each basic substratum (table 3*a*) was further divided into specific habitats (table 3*b*), based on the distinctions made in most trophic models [22] and each food category was assigned to an abundance interval (1–3), for each of 10 specific habitats (table 3*b* and figure 2), as described above. For example, hard substrata were grossly divided into rocky reefs and caves, according to the different exposures to light and external influences characterizing these environments. Similarly, soft substrata were divided into open sand and embayments, based on variable shelter influencing plant and animal communities (table 3*b*).

**Table 3.** Computation of RAFI. The abundances of each trophic group (in columns), referring to substrata and habitats (in rows), are derived from the available literature (electronic supplementary material, table S1). (*a*) Trophic resource abundances in relation to basic substrata. (*b*) Trophic resource abundances in relation to specific habitats. The considered trophic groups are: microcarnivores (mCa), carnivores (Ca), microherbivores (mHe), herbivores (He), microomnivores (mOm), omnivores (Om), microdetritus feeders (mDeF), detritus feeders (DeF), detritus feeders–suspensivores (DeFS), Detritus feeders–herbivores (DeFHe) and filter feeders (FF).

(*a*) basic substrata		mCa	Ca	mHe	He	mOm	Om	mDeF	DeF	DeFS	DeFHe	FF
soft substratum		1	1	1	1	2	2	1	1	2	1	1
hard substratum		1	1	1	2	2	2	2	2	2	1	1
hard biogenic		2	1	1	2	2	2	2	1	2	2	2
macroalgae beds		1	1	1	3	1	2	2	2	2	2	1
seagrass beds		2	1	1	3	2	2	2	3	2	3	1

(*b*) basic substrata	specific habitats	mCa	Ca	mHe	He	mOm	Om	mDeF	DeF	DeFS	DeFHe	FF
soft	sand	1	1	1	2	1	1	1	2	1	1	1
	embayments	2	1	2	3	1	1	3	2	3	3	3
hard	rocky reef	2	2	2	3	2	1	2	2	3	3	1
	caves	1	1	1	1	2	1	1	1	2	1	1
hard biogenic	coral reefs	1	2	1	1	2	2	2	1	2	2	2
	coralligenous	2	1	2	3	2	1	2	2	2	2	2
macroalgae	kelp	2	2	2	2	2	1	2	2	3	3	1
	fucoid	3	3	2	3	2	1	2	1	2	2	2
seagrass	low-canopy seagrass	2	2	2	2	2	3	2	2	1	1	1
	high-canopy seagrass	3	3	3	3	2	3	2	3	3	3	2

Each ecosystem was consequently classified according to the amount of food potentially available to each trophic group (tg), according to the following relationship:

2.1 $${\text{Resource abundance}}_{\left(\mathrm{tg},\mathrm{ecosystem}\right)}=f(\text{basic substratum}\times \text{specific}\;\text{habitat})$$

This permits estimation, for example, that the plant standing stock potentially available for herbivores (He) is maximum in a fucoid or a seagrass meadow, lower in harbours and lowest on sandy substrata, coral reefs and caves (table 4*a*).

**Table 4.** (*a*) Final scores with RAFI predictions for average abundances of trophic resources in each habitat. (*b*) Modifiers for local conditions. Trophic groups: mCa (microcarnivores); Ca (carnivores), mHe (microherbivores), He (herbivores), mOm (microomnivores), Om (omnivores), mDeF (microdetritus feeders), DeF (detritus feeders), DeFS (detritus feeders--suspensivores), DeFHe (detritus feeders–herbivores) and FF (filter feeders).

(*a*) habitats	mCa	Ca	mHe	He	mOm	Om	mDeF	DeF	DeFS	DeFHe	FF
sand	1	1	1	2	2	2	1	2	2	1	1
embayments	2	1	2	3	2	2	3	2	6	3	3
rocky reef	2	2	2	6	4	2	4	4	6	3	1
caves	1	1	1	2	4	2	2	2	4	1	1
coral reef	2	2	1	2	4	4	4	1	4	4	4
coralligenous	4	1	2	6	4	2	4	2	4	4	4
kelp	2	2	2	6	2	2	4	4	6	6	1
fucoid	3	3	2	9	2	2	4	2	4	4	2
low-canopy seagrass	4	2	2	6	4	6	4	6	2	3	1
high-canopy seagrass	6	3	3	9	4	6	4	9	6	9	2

(*b*) modifiers	mCa	Ca	mHe	He	mOm	Om	mDeF	DeF	DeFS	DeFHe	FF
exposed	0.8	1	1	0.8	0.5	1	0.4	0.7	0.4	0.8	0.4
sheltered	1.2	1	1.5	1.2	1	1.2	1	2	1.2	1.5	1
eutrophic	2	2	1.2	1.5	1	1	1.2	1.6	2	2	1.2
oligothrophic	3.3	3	2.2	3.5	3	2.8	2	1	0.8	2.3	1
anthropogenic perturbations	1	1.2	1.5	1.2	2	2	2	3	3	2.2	3.2
natural perturbations (e.g. estuaries)	1.1	1	0.6	1.1	1.1	2	3	3	1.8	2	2
deep	1.2	1.1	0.6	0.6	1	1	1.1	1.1	0.9	1.1	0.9
shallow	1.5	0.8	3	3	0.8	1.5	1.6	1.8	1.5	2	0.8

Finally, modifying factors were considered, to explain how local environmental conditions influence the food resources available for a particular trophic group with respect to the average conditions for a given habitat. These modifiers acknowledge that other factors, besides the type of substratum and the specific habitats, influence the community composition and the abundance of trophic resources [20,23,24]. For example, variations of light irradiance owing to depth may dramatically influence the abundance of plant biomass present in a deep rocky reef or seagrass meadow. The abundance of epiphytes in a shallow *Posidonia oceanica* meadow is approximately three times that recorded in a deep meadow [25]. Also, the abundance of organic detritus available for detritivore consumers is largely influenced by exposure to waves and currents [26]. Eutrophic and oligotrophic conditions influence the standing crop of primary producers, even when the same ecosystem is considered [27]. Therefore, the relative food resources estimated for each habitat (table 4*a*) must be tuned according to these site-specific influences (table 4*b*) and the relationship (2.1) is set as:

2.2 $$\text{Resource}\;\text{abundanc}{\text{e}}_{\left(\mathrm{tg},\mathrm{ecosystem}\right)}=f(\text{basic}\;\text{substratum}\times \text{specific}\;\text{habitat})\times \text{specific}\;\text{modifiers}$$

For this purpose, literature data were screened to detect deviations from ‘average’ expected conditions under the influence of each modifier. A value of 1 was set for each trophic category under standard conditions (table 4*b*), meaning that the estimate of food resources, obtained in table 4*a*, will not change. In contrast, exposure to modifying conditions will increase or decrease the relative amount of food resources available. For example, higher currents induce a mean decrease of 20% for the food resources available for mCa, as determined by screening the results of studies comparing similar ecosystems exposed to different strength currents [28]. Therefore, a modifying value of 0.8 was assigned in this case (table 4*b*).

Some modifiers produce dramatic variation from average conditions. Food resources available for mCa may be surprisingly high (330%) in oligotrophic systems [29,30], while other trophic resources (e.g. DeF and FF) are not influenced. This is reflected in the modifying value of 3.3 in table 4*b*, corresponding to the trophic resources mCa in oligotrophic environments.

These modifiers are applied only where documented local conditions strongly influence the relative availability of trophic resources in the considered habitats. We considered ‘shallow’ habitats those in water less than 5 m deep, and ‘deep’ habitats those located below a depth of 25 m. We considered ‘exposed’ those ecosystems open to large sea swells or characterized by very high winds, and ‘anthropogenically impacted’ those systems for which there are clear and documented evidence for major industrial, fishery or urban pressures. Thus, only a few characterizing pressures—the most evident and well documented—are considered for each site (see grey cells of table 5*a*), to avoid interference with the basic environmental features of ecosystems.

**Table 5.** (*a*) Classification of sites used for testing. Each site used to test the model performances is classified according to its habitat type (X, yes; white cells) and modifying conditions (X, yes; grey cells). (*b*) RAFI calculated for each of the above test sites (table 5*a*). For example, in the case of mCa in the low-canopy seagrass San Pietro, the RAFI estimate for low-canopy seagrass (4) is multiplied by the modifiers for ‘Eutrophic’ (2) and ‘Shallow’ (1.5) to obtain a final RAFI score of 12, which is then converted to RAFI% as shown in Table 5*c*. The original data and computations are available in electronic supplementary material, table S2. (*c*) Relative abundance of food items (RAFI%) calculated for each of the test sites based on table 5*b*. For example, in ‘San Pietro’, the trophic resources (relative abundance of food items) available for microcarnivores are 11% of the total trophic resources in this system, whereas the resources available for carnivores account only for 3% of the total resources of the ecosystem.

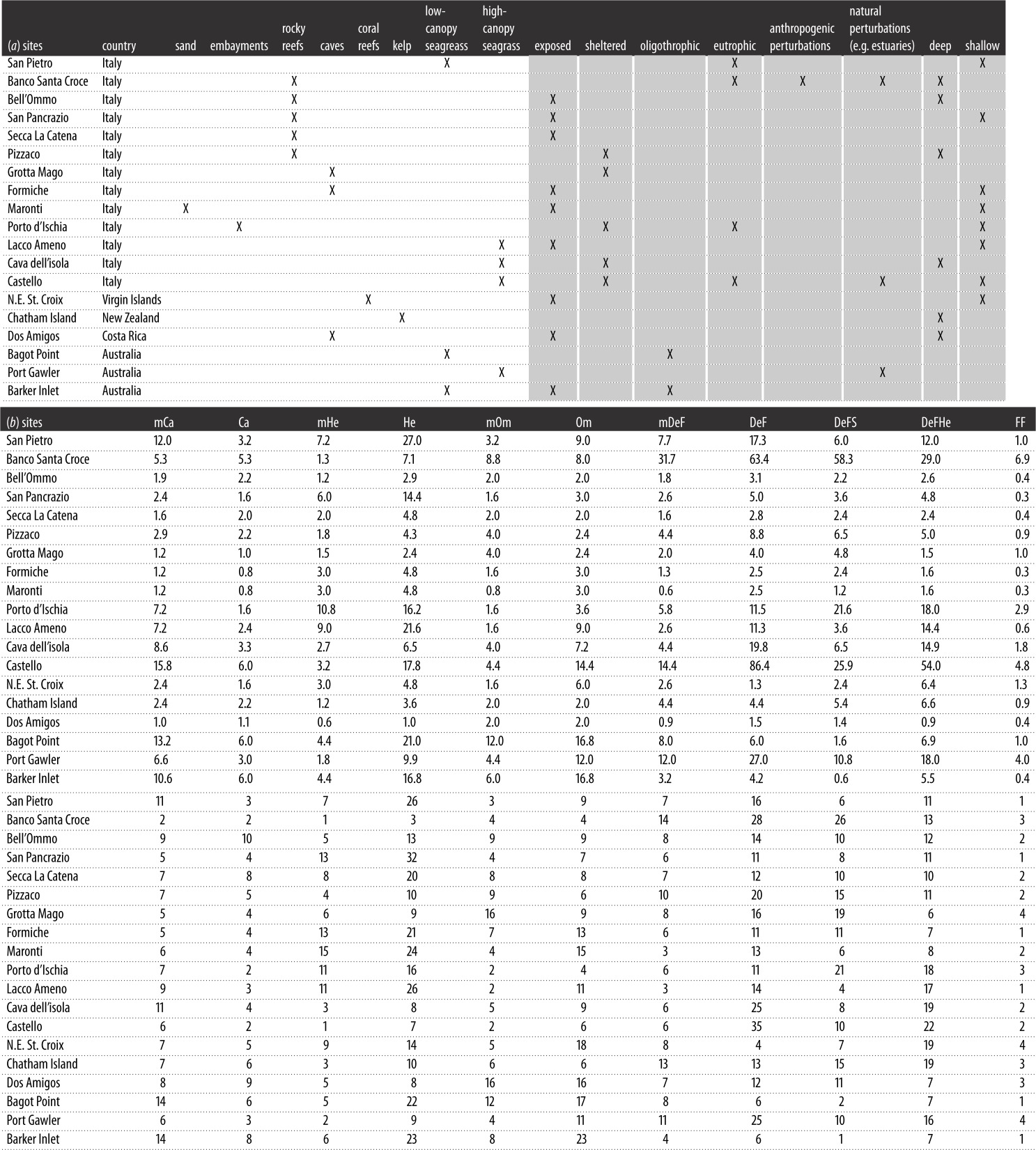

### 2.2. Application of relative available food index tables

To test the effectiveness of simulations provided by RAFIs, 19 different sites were chosen throughout the world, among those for which sufficient information was provided on the abundance of food items (permitting at least partial comparisons between computed and actual data). In fact, most studies provide incomplete sets of trophic groups and, in this case, comparisons with the whole trophic model provided by RAFI is not feasible. In particular (table 5*a*), each site (in rows) was classified according to its characteristics (in columns). The site descriptors (in each line) were set to ‘X’ when that specific feature was applicable, and left blank (null) when the feature was not applicable (table 5*a*). For example, ‘San Pietro’ (the site reported in the first row) is a eutrophic (fourth grey column), shallow (last grey column) environment in the bay of Naples (Italy), hosting a low-canopy seagrass (*Cymodocea nodosa*). In contrast, ‘N.E. St. Croix’ (the site reported in the 14th line) is a shallow, exposed coral community in the US Virgin Islands. Each site was similarly characterized.

This classification permitted the computation of the abundance of food items (table 5*b*), according to the above-described RAFIs. For example, in the case of ‘San Pietro’, the values for each trophic category were computed by multiplying all the scores previously marked with ‘X’ in table 5*a*, i.e. the scores in line 8 of table 4*a* (low-canopy seagrasses) by the scores in lines 3 and 8 (eutrophic and shallow, respectively) of table 4*b*, following the relationship (2). The same computation was performed for all the other considered sites (electronic supplementary material, table S2), according to their environment type and local specific pressures, as reported in the literature. Repeating this procedure, the scores for each trophic category in each site were computed (table 5*b*). These computations are available in digital format in electronic supplementary material, table S2, along with an empty spreadsheet to be used for the simulation of further datasets.

Finally, the values in each cell were converted, line by line, to a percentage of the total resources present in each site (RAFI%), in order to standardize the results and make them comparable among different ecosystems [31]. Thus, RAFI% (table 5*c*) allows comparisons among such different ecosystems as coral reefs, temperate harbours, seagrass meadows and sand bottoms, which are characterized by wide ranges of densities of organisms, dynamics and productivities.

For testing the trophic resources at three additional Australian sites (Bagot Point, Port Gawler and Barker Inlet), only the abundance of the resources for three major trophic groups (He, DeF and Ca) was reported in the literature [32]. Therefore, the per cent contributions of trophic resources for He (leaf biomass), DeF (debris biomass) and macrocarnivores (fauna greater than 1 mm), as found in the literature, were compared with the same food resources predicted by RAFI (table 6).

**Table 6.** Comparison of trophic resources reported by Edgar & Shaw [32] for three Australian sites (top part) with the results of RAFI predictions (bottom part). The proportion of trophic resources among the three main trophic groups for which experimental data were available has been calculated. Their percentages (% proportion of biomass versus RAFI%) are compared (right part of the table).

	actual biomass (g m^−2^)			% proportion of biomass reported		
Australian sites (as reported in the literature)	leaf	debris	fauna >1 mm	leaf	debris	fauna >1 mm
Bagot Point (*Zostera* sp.)	47	3.23	17	69.9	4.8	25.3
Port Gawler (*Posidonia* sp.)	206	332	60	34.5	55.5	10
Barker Inlet (*Heterozostera* sp.)	199	n.d.	45	81.6	n.d.	18.4

	RAFI predictions (abundance units)			RAFI% predictions: resources for		
model predictions	He	DeF	Ca	He	DeF	Ca
Bagot Point (*Zostera* sp.)	21.7	6.2	6.2	63.6	18.2	18.2
Port Gawler (*Posidonia* sp.)	9.9	24.7	2.7	26.5	66.1	7.4
Barker Inlet (Heterozostera sp.)	22.5	5.6	8.1	62.2	15.6	22.2

Finally, a simulation for a marine protected area (MPA) in Africa, for which some literature information is available [33], was performed in order to test the sensitivity of the method for computing changes occurring after the institution of the protection plan. In this case, the factor ‘anthropogenic perturbations’ was set to ‘X’ before the institution and ‘null’ after the institution, to perform the simulation (electronic supplementary material, table S2).

RAFI tables were formally validated by comparing observed food resources to those predicted. For this purpose, two comprehensively sampled sites were considered: Lacco Ameno [34] and Banco di Santa Croce [35]. These sites were selected because (i) complete datasets were available and (ii) they host quite different environments (table 5*a*): seagrass versus hard bottom, eutrophic versus pristine, shallow versus deep, etc. Fauna was sampled using an airlift sampler [35] in two replicate 40 × 40 cm surface area plots, and all specimens collected were counted and identified at the species level.

Lacco Ameno (40°45′ N, 13°53′ E) is located in the northwest sector of the Island of Ischia (Bay of Naples, Italy). It contains a continuous and dense meadow of *P. oceanica* extending from 1 m to about 33 m (deep limit). Samples collected at a depth of 5 m were considered. Animals were grouped according to their possible role as prey for macrocarnivores, microcarnivores, filter feeders, etc. Data were integrated, when necessary, with gut content analyses evaluated for each sampled species. Prey item size was taken into account and their abundance in the environment was evaluated based on the following relationship:

2.3 Total food biomass available=number of items×average individual biomass

The abundance of food available for macroherbivores and microherbivores and the actual abundance of detritus were evaluated according to [36]. The results obtained were transformed into % abundance of each food item and compared with the abundance of food items (RAFI) computed according to table 4.

Banco di Santa Croce (40°40′ N, 14°26′ E) is a submerged seamount complex located in the eastern Gulf of Naples. It is located 0.8 km off the coast and is composed of various rocky seamounts arising from a depth of 60 to 11 m, forming a circular structure. Samples were obtained over a 3 year extensive sampling programme to develop a trophic model for the site [37]. Direct measurements provided the actual abundance of food items and the abundance of species of each trophic group per square metre. The total number of individuals per m^2^, as well as the total biomass of each trophic group and abundance of organic detritus and of phyto- and zooplankton were also available [37], and converted into the same units to allow direct comparisons. The fish fauna was surveyed using visual census [37].

### 2.3. Statistical analyses

The *r^2^* coefficient was calculated using correlation analysis to evaluate how well the RAFI predictions for each trophic group fitted data for the selected sites derived from the literature. The results were confirmed by the *G*-test (likelihood ratio test).

The actual data sampled in the two validation sites were compared with the patterns of abundance of resources obtained by means of our model, and *t*-tests were used to determine the significance of the difference of the slope from the null hypothesis of a 0 slope using GraphPad Prism 4 (GraphPad Software, San Diego, CA). Pearson's product–moment correlations were also used to test agreement between RAFI estimated and observed food resources at the sites for which complete data across all trophic groups were available. For all the other sites, RAFI predictions were qualitatively compared with the available literature data, even when incomplete, by detecting the dominant food resources predicted by RAFI and their correspondences with the dominant food resources described in the literature.

## 3. Results

### 3.1. Relative available food index validation

**Figure 3.**
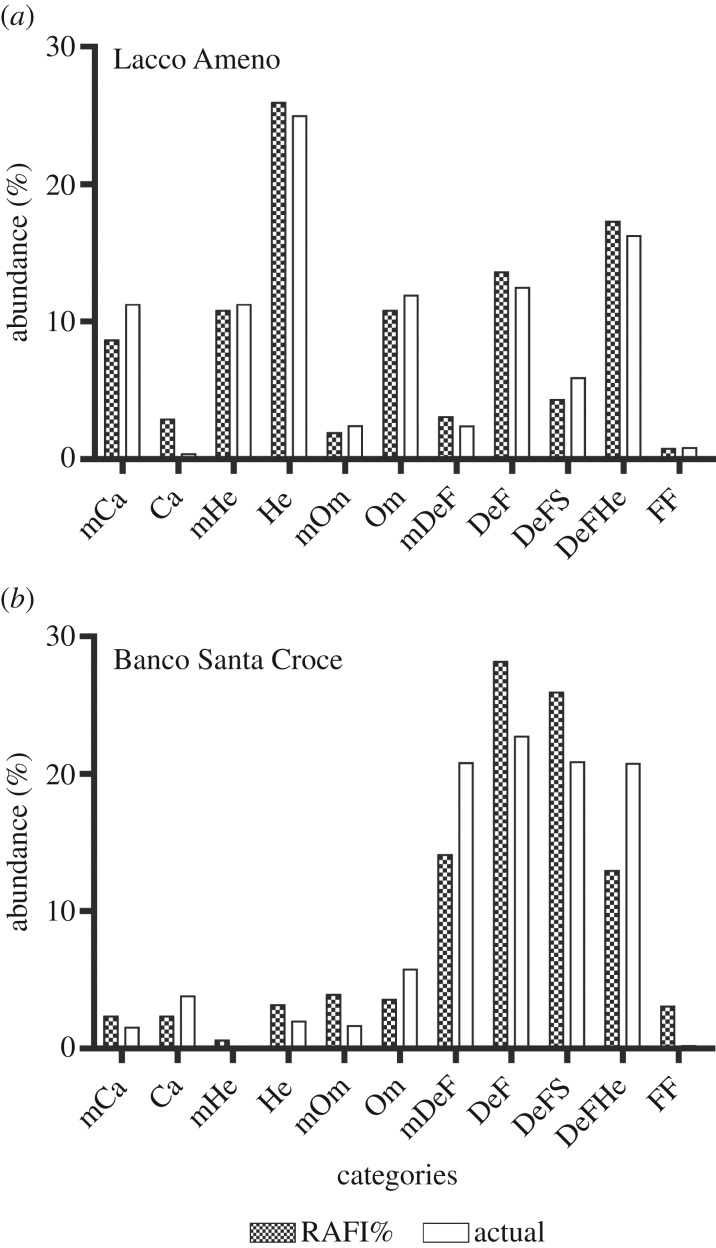
(*a*) RAFI simulation and actual per cent abundance of resources available for various feeding groups, obtained for Lacco Ameno (Ischia Island, Italy); (*b*) RAFI predictions and actual per cent abundance of resources available for various feeding groups, obtained for Santa Croce Bank (Bay of Naples, Italy).

**Figure 4.**
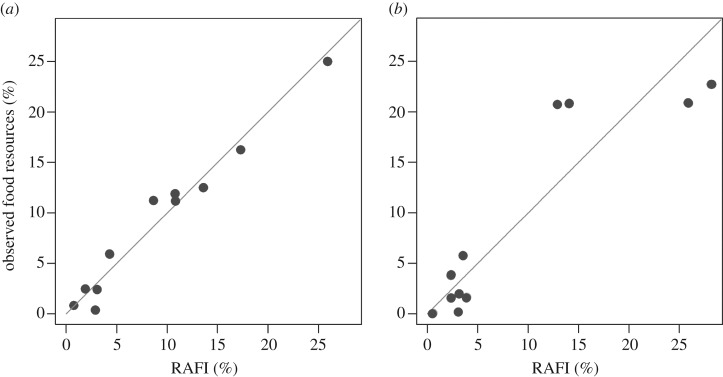
Observed values of per cent abundance for trophic resources versus RAFI% estimated values for resources present in two Mediterranean sites. The grey line denotes 1 : 1 agreement between the two methods. (*a*) Lacco Ameno; *t* = 15.72, d.f. = 9, *p*-value <0.001, *r^2^* = 0.97. (*b*) Banco di Santa Croce; *t* = 6.38, d.f. = 9, *p*-value <0.001, *r^2^* = 0.82.

The comparison of the abundances of food items estimated by means of the proposed method with field data shows some differences, but trends coincide (figure 3). In particular, data for Lacco Ameno d'Ischia (figure 3*a*) show good agreement between RAFI% simulated data and observed data, other than carnivores (Ca), which appear to be overestimated by RAFI. As for the other trophic categories, herbivores, DeF and DeFHe, as well as mDeF, are slightly higher when calculated by RAFI, whereas mCa, mHe, Om and DeFS are slightly lower than actual. The most abundant resource is macroherbivore food, accounting for about 25% of the total trophic resources available, followed by DeFHe (about 15%), omnivores, mHe and mCa (about 10%). On the whole, the relationship between actual and RAFI estimated data was highly significant (figure 4*a*, *r^2^* = 0.97).

In the case of Banco di Santa Croce (figure 3*b*), field data show fundamentally two types of trophic categories: those relying on low abundance resources (mCa, Ca, mHe, He, mOm, Om and FF) and those relying on locally abundant resources (mDeF, DeF, DeFS, DeFHe). RAFI predictions respect this pattern (*r^2^* = 0.82 between predicted and field data; figure 4*b*), apart from some variability observed in individual categories.

**Figure 5.**
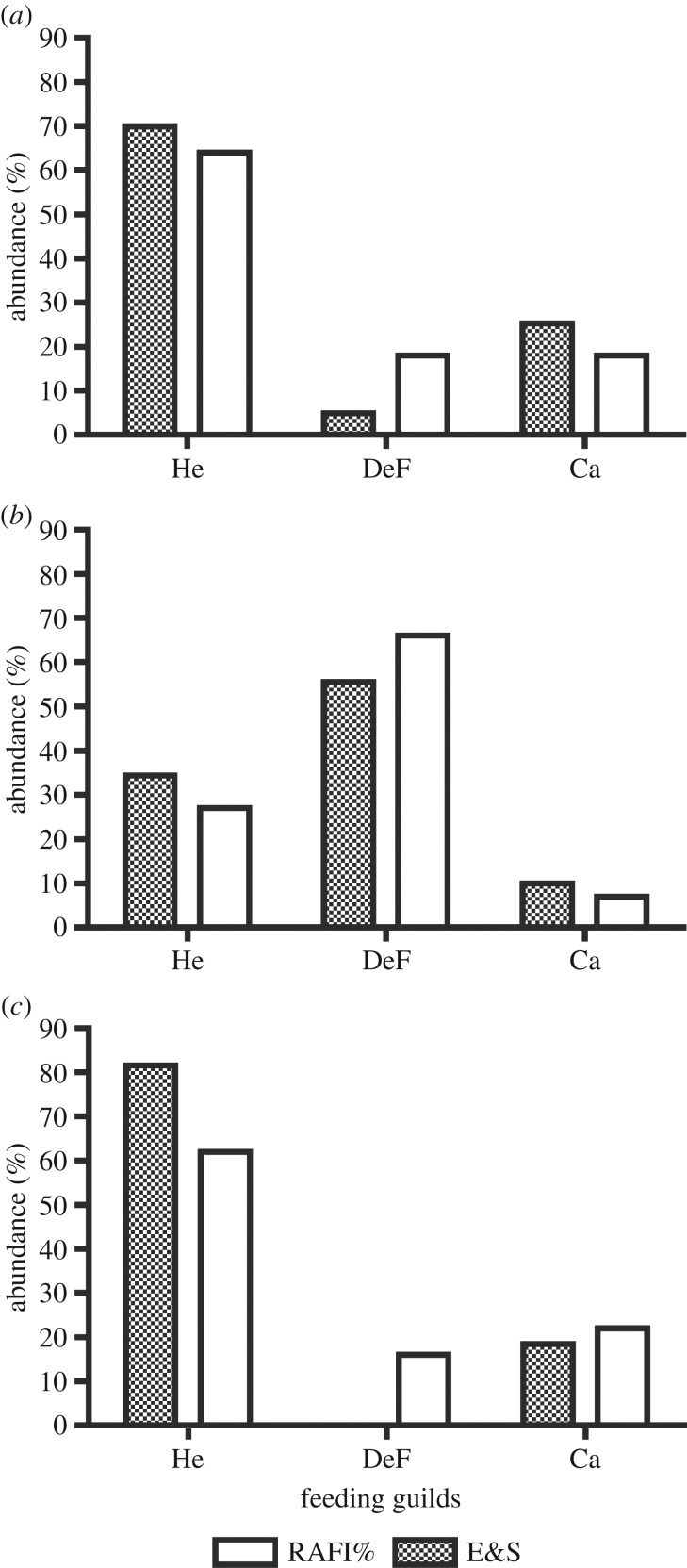
Comparison of the results reported by Edgar & Shaw [32] on the abundance of trophic resources for He, DeF and Ca. Edgar & Shaw [32] data (E&S) are indicated by grey bars, against predictions of the RAFI model (RAFI%, white bars). Three sites are considered, for which sufficient literature data were available: (*a*) Bagot Point, (*b*) Port Gawler and (*c*) Barker Inlet.

Similarly, *t*-tests indicated no significant differences (*p* < 0.001) between the RAFI data simulated for three Australian sites hosting seagrass meadows and field data, according to the known feeding groups investigated (table 6 and figure 5). In addition, data reported in the literature on the abundance of the main trophic groups were compared with the results of RAFI predictions for various sites (table 7), with good coincidence.

**Table 7.** Comparison of predicted RAFI% and abundance of trophic resources derived from the available literature. For each site, the most abundant trophic groups identified by RAFI% are indicated in the second column. The most abundant trophic group (TG) or trophic resources (TR) reported for each site in the literature (fourth column) are provided in the third column. Country abbreviations are Italy, IT; United States Virgin Islands, US; Costa Rica, CR; New Zealand, NZ.

site	RAFI-predicted highest trophic resource(s)	most abundant trophic resources (TR) or trophic group (TG) according to the literature	references (electronic supplementary material, table S1)
San Pietro (IT)	He	herbivorous molluscs (TG)	[131]
San Pietro (IT)	DeF	detritivorous polychaetes (TG)	[132]
Bell'Ommo (IT)	DeF, DeFS	gorgonians (TG)	[133]
San Pancrazio (IT)	He, mHe	algae (TR)	[134]
Secca La Catena (IT)	He, DeF	algae (TR)	[135]
Pizzaco (IT)	He	algae (TR)	[135]
Pizzaco (IT)	DeF, DeFS, DeFHe	gorgonians (TG)	[135]
Grotta del Mago (IT)	DeF	detritus feeding amphipods (TG)	[136]
Grotta del Mago (IT)	DeFS	sponges (TG)	[137]
Formiche (IT)	He	algae (TR)	[138]
Formiche (IT)	mHe	diatoms (TR)	[138]
Formiche (IT)	Om	both animal and algae associations (TR)	[138]
Maronti (IT)	DeF, mHe	detritus and plant material (TR)	[139]
Maronti (IT)	He	drift algae (TR)	[140]
Porto d'Ischia (IT)	DeF	organic detritus (TR)	[141]
Porto d'Ischia (IT)	He	algae (TR)	[142]
Cava dell'Isola (IT)	DeF	seagrass and detritus (TR)	[143]
Castello (IT)	DeF	sea urchins (TG)	[144]
Castello (IT)	He	herbivorous fishes (TG)	[145]
Castello (IT)	DeFHe	DeFHe (TG)	[146]
N.E. St. Croix (US)	He, DeFHe	herbivores (TR)	[147]
Chatham Island (NZ)	mDeF, He	detritus feeders and herbivores (TG)	[148]
Dos Amigos (CR)	Om, DeF	DeF and Om echinoderms (TG)	[149]

**Figure 6.**
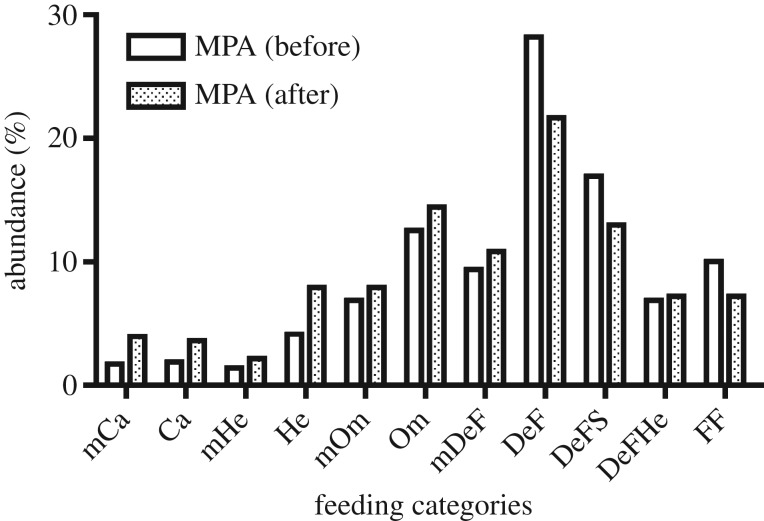
RAFI simulation for an MPA in Sine Saloum (Senegal), before and after the institution of the no-take area. The % abundances of trophic resources available for each feeding category are reported. The area is composed of a ‘sand’ bottom and contains some ‘natural perturbations’. Therefore, these two indicators were set to ‘X’ in the classification of the sites. In addition, to simulate the local food webs before the MPA institution, the indicator ‘anthropogenic perturbations’ was set to ‘X’; to simulate the local food webs after the institution of the MPA, the indicator ‘anthropogenic perturbations’ was shifted to ‘nil’. The corresponding scores (table 4*a,b*) were multiplied according to the relationship (2.2).

Finally, the simulation of the Sine Saloum MPA [33] produced clear differences before and after the institution of the protection plan. In particular (figure 6), the resources available for microcarnivores, carnivores, herbivores and omnivores showed an increase in the protected conditions, whereas the trophic resources available for detritus feeders and herbivore–detritus feeders exhibited a decrease after the institution of the MPA (i.e. in the absence of ‘anthropogenic influences’).

### 3.2. Test of relative available food index in various sites of the world

**Figure 7.**
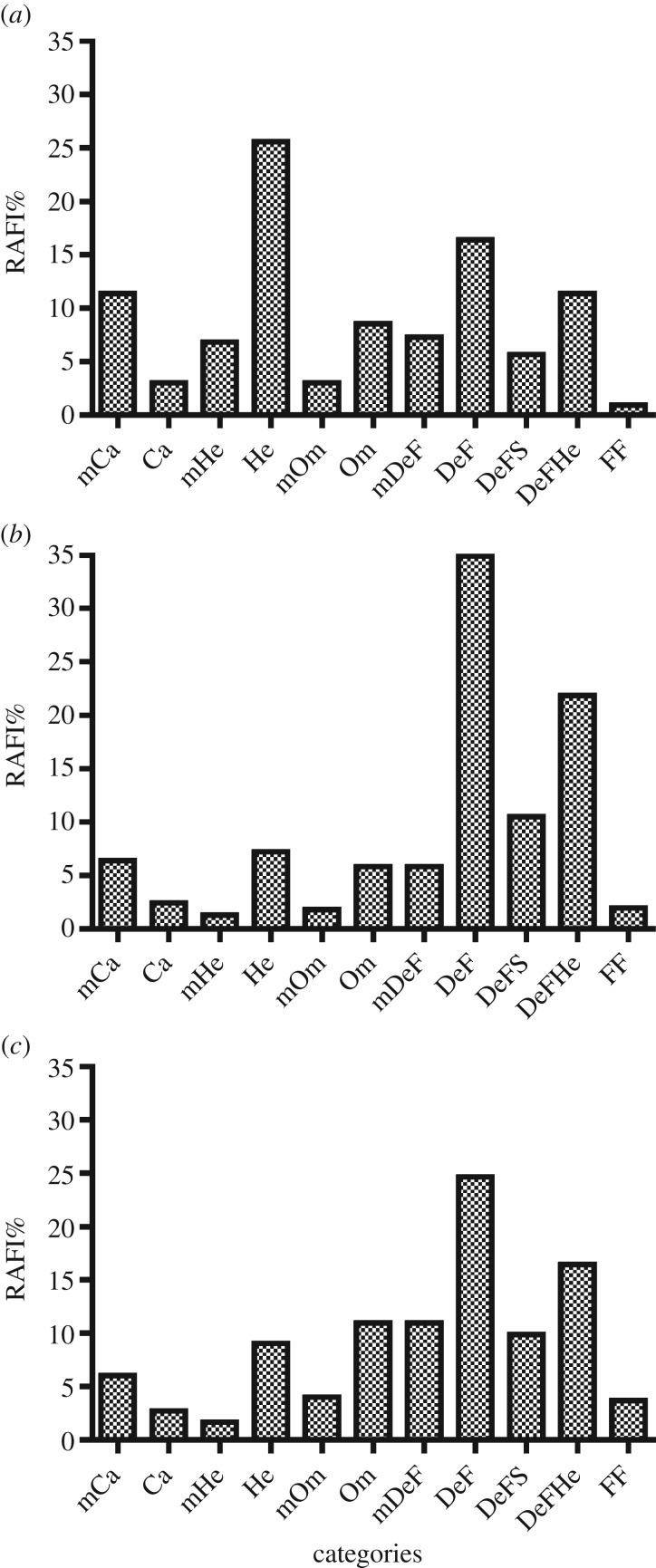
Distribution of trophic resources (expressed as RAFI%) for three selected sites containing seagrasses: (*a*) San Pietro, *Cymodocea nodosa* meadow in the Bay of Naples; (*b*) Castello, *Posidonia oceanica* meadow established in a highly acidified area off the island of Ischia (Italy); (*c*) Port Gawler, *Posidonia* sp. meadow in Australia.

The trophic resources available at various sites were predicted by RAFI and clear distinctions were obtained, according to specific ecological conditions, even when similar ecosystems were considered. Comparing the trophic resources available in three sites hosting seagrass meadows (San Pietro, Castello, Port Gawler), we observed very different patterns of resource distribution (figure 7). In San Pietro, which hosts a low-canopy seagrass bed (*C. nodosa*), most trophic resources are available for herbivores (26%), followed by detritus feeders (16%), detritus feeder–herbivores and microcarnivores (11%). In contrast, in Castello d'Ischia, an acidified site hosting a high-canopy seagrass (*P. oceanica*), most trophic resources are available for detritus feeders (35%), followed by DeFHe (22%) and DeFS (10%). The Australian Port Gawler site hosts a *Posidonia* sp. meadow and exhibits maximum abundance of resources for detritus feeders (25%) followed by DeFHe (16%) and DeFS (10%), showing the importance of plant detritus in this Australian seagrass ecosystem.

### 3.3. Relative available food index trends in various environments

RAFI computations indicated that trophic resources available for mCa reach highest abundance in several seagrass environments, coralligenous and fucoid habitats, and lowest abundance in rocky bottoms and caves. Similarly, trophic resources available for herbivores (He) reach maximum abundance in seagrass meadows and in shallow rocky bottoms, while they dramatically decrease in deep rocky bottoms and caves (table 5*c*). The abundance of resources available for omnivores (Om) is minimum in rocky bottoms and embayments, while detritus feeder resources (DeF) are relatively abundant in high-canopy seagrasses, caves and rocks. Finally, the abundance of resources available for FF is generally low and sensitive to the effect of specific modifiers, in the considered environments. In fact, according to RAFI, the abundance of food available for FF accounts for 4% of the total trophic resources in some caves (Grotta del Mago), and in an analogous environment (Formiche) it declines to 1% of the total trophic resources.

## 4. Discussion

### 4.1. The accuracy of model predictions

The availability of individual food resources in shallow marine ecosystems varies with environmental features [31], but the data published on the arrangement of resources in each ecosystem are generally incomplete and not comparable. The proposed model represents a framework to predict the relative abundance of food resources for trophic groups present in marine areas, based on the habitat considered and local specific influences (e.g. high currents, low depth, etc.). We demonstrated that the model predictions agree well with the trophic data reported in studies undertaken in a wide range of ecosystems, both temperate and tropical.

Statistical comparisons between RAFI-predicted and observed trophic resources at two intensively studied Mediterranean sites demonstrate the accuracy of the RAFI estimates. RAFI predictions for Lacco Ameno are in close agreement with measured abundances of trophic resources. The large abundance of trophic resources potentially available for herbivores at this site was expected, since this is a *P. oceanica* environment, represented by a dense meadow exhibiting a leaf standing stock peaking at 340 g dry weight per square metre [36]. RAFI provides an appropriate estimate of the large biomass potentially available for macroherbivores. However, relatively little of this biomass is directly consumed by grazers, owing to various deterrent compounds [38,39]. Only a few herbivores, sometimes reaching high densities, are able to consume the abundant green leaf biomass, most notably sea urchins [40], some isopods [41] and a few fishes [42]. As predicted, modelled food availability does not necessarily correspond to food consumption.

The RAFI model, in fact, predicts available biomass, not consumption, and individual consumers may exploit the available resources at various levels, according to their abilities for fragmenting and detoxifying food items [43]. Consequently, the abundance of resources estimated by RAFI represents the potential abundance of food accessible for each category of consumers, and is independent of the ability of individual species to exploit the resource (top-down control).

The second most abundant food resource in Lacco Ameno, based on both RAFI and observed data, is for DeFHe. Fundamentally, this is plant detritus, which is very abundant in *P. oceanica* meadows [44,45] and, in particular, in Lacco Ameno, where 42% of the plant primary production is transformed into detritus that is degraded *in situ* [36]. This large biomass is available for several consumers, including crustaceans and some echinoderms [46].

A divergence between RAFI and observed data at Lacco Ameno was found for macrocarnivores (Ca). However, ‘macrocarnivores’ in *P. oceanica* ecosystems are principally represented by fishes [47], which often consume other fishes [48,49], whereas invertebrate macrocarnivores are present only in the rhizome layer and they are represented by a few species of large decapods and echinoderms [50]. Interestingly, literature data on fish stocks could not be considered for the evaluation of the actual biomass, since the methods applied for their collection in this site did not refer to a surface area [51]. In contrast, the abundance of other trophic resources was evaluated on a surface unit base, according to the literature [17,34,36]. If the fish fauna was considered and added to the actual abundance of resources for carnivores, this value would increase substantially. Thus, RAFI arguably provides a more reliable value for the abundance of carnivore trophic resources than data obtained from the literature, because the abundance of fish per surface unit was not precisely evaluated through field investigations.

This outcome emphasizes the need for development of a general model to estimate trophic resources. RAFI estimates trophic resources of ecosystems while avoiding methodological constraints hindering comparison of food resources measured with different scales or units. In fact, owing to methodological constraints, researchers generally consider only a subset of trophic resources, which can lead to incorrect conclusions when different environments are compared.

### 4.2. Further validation on a rocky environment

The RAFI estimates for Banco di Santa Croce indicate two distinct categories of trophic resources: those present in low abundance (less than 5% of total trophic resources), such as those sustaining populations of carnivores, herbivores and omnivores, and those present in large abundance, all linked to the organic detritus. Food webs in this rocky area are mostly established on the organic detritus deriving from Sarno River [52,53] and a good statistical match between actual data and RAFI estimates was demonstrated.

The largest difference between RAFI-predicted and observed trophic resources at Banco di Santa Croce was in the resources available for FFs. However, this particular site is characterized by an exceptional biodiversity and abundance of FFs (sponges, gorgonians, corals, etc.), which together must rapidly deplete available trophic resources [37]. Therefore, the abundance of food for FFs, as sampled, is potentially low, owing to rapid consumption by animals according to a very strong top-down control of their abundance. In this case, the RAFI value, indicating the abundance of resources potentially available for these organisms, could be closer to an index of production.

Throughout this study, we have considered food abundance as a proxy for production because very few studies describe production for a range of food items. Nevertheless, at locations with rapid turnover of particular dietary items this assumption may introduce over-prediction, compared with measured values [5] of standing stocks. The actual abundance of trophic resources measured in the field (i.e. their standing stocks) is determined by bottom-up control (the amount of production) as well as top-down control, owing to the activity of consumers. Therefore, measured divergences from the RAFI model of resource distribution might be used to improve our understanding of real ecosystems, the effects of human disturbances, the propensity of ecosystems to be invaded and their overall stability as a result of boom and bust dynamics at given trophic levels.

### 4.3. Relative available food index tested at sites in the world

The RAFI tables computed in this study demonstrated good predictions of the relative trophic resources available to each trophic group in the ecosystems tested, coinciding with the most abundant trophic resources, or the trophic groups feeding on them, at several coastal sites worldwide (table 7). Also, the sensitivity exhibited in the simulation of the MPA in Senegal (Sine Saloum Delta) is remarkable. In fact, a specific investigation [33] found, as a consequence of the MPA institution, a decrease in the abundance of herbivore–detritivore fish (from 44.0% to 6.3% in biomass), and a decrease in FF–microplanktivore fish (from 31% to 12.5% in biomass) when compared with a significant increase of carnivore and omnivore fish (from 5.9% to 49.6% and from 5.2% to 11.8%, respectively). These data are in accordance with the scenarios provided by RAFI, indicating a clear decrease of trophic resources for DeF and FF, and an increase of resources available for Ca and Om, although our computations pertain to the whole food webs (including all animal taxa) of the area, whereas the data available in the literature are referred to the fish compartment only. The total biomass of consumers is related to the abundance of their trophic resources [6]. Therefore, a general agreement between the estimated available resources and the actual abundance of their consumers was found, although published data are insufficient for formal comparisons.

RAFI tables require further tests to extend the general applicability of the proposed model to other ecosystems [31]. Nevertheless, the RAFI framework developed to describe the trophic resources available in specific habitats and the modifying effect of local conditions can now be applied and tested in any natural ecosystem worldwide.

## Supplementary Material

Table S_1. Literature used to rank the abundance of trophic resources among ecosystems. The last section (biotope typologies) has been used principally to detect natural or anthropogenic pressures influencing the basic patterns of food availability. Reference numbers indicated in Tables 1 and 6 refer to the papers listed here.

## Supplementary Material

Table S_2. a) Original spreadsheets used to evaluate RAFI in the ecosystems considered in the manuscript; b) User spreadsheets to be filled with own data for computing the Relative Abundance of Trophic Resources (RAFI) in other ecosystems. Please fill only the white cells: other cells must not be modified. Do not consider empty stations: they all result equal to "1.0" due to lack of data

## Data accessibility

All data and supplementary material are available at the Stazione Zoologica Anton Dohrn and in the Dryad Digital Repository: http://dx.doi.org/10.5061/dryad.nt91j [54].
