# Supplementary material for: Relating trophic resources to community structure: a predictive index of food availability
Source: R Soc Open Sci. 2017 Feb 8;4(2):160515. doi: 10.1098/rsos.160515 (PMC5367299; doi:10.1098/rsos.160515)
Supplement: Table S_1. Literature used to rank the abundance of trophic resources among ecosystems. The last section (biotope typologies) has been used principally to detect natural or anthropogenic pressures influencing the basic patterns of food availability. Reference numbers indicated in Tables 1 and 6 refe [file rsos160515supp1.docx]

**Electronic Supplementary Material**

**Table S_1.** Literature used to rank the abundance of trophic resources among ecosystems. The last section (biotope typologies) has been used principally to detect natural or anthropogenic pressures influencing the basic patterns of food availability. Reference numbers indicated in Tables 1 and 6 refer to the papers listed here.

**Marine Caves**

1. Beron P, Petrov B, Stoev P. The invertebrate cave fauna of the Western Rhodopes (Bulgaria and Greece). The invertebrate cave fauna of the Western Rhodopes (Bulgaria and Greece). — In: Beron, P. (ed.). Biodiversity of Bulgaria, 4. Biodiversity of Western Rhodopes (Bulgaria and Greece). Pensoft & Nat Mus Natur Hist, Sofia. 2011; pp. 583—661.
2. Chimenz-Gusso C, Fresi E, Cinelli F, Mazzella L, Pansini M, Pronzato R. Evoluzione dell ebiocenosi bentoniche di substrato duro contro un gradient di luce in una grotto marina superficiale. II Pantopodi. Mem Biol Mar Oceanogr. 1978; VIII: 91-103.
3. Cinelli F., Fresi E, Mazzella L, Pansini M, Pronzato R, Svoboda A. Distribution of benthic phyto- and zoocoenoses along a light gradient in a superficial marine cave. In: Keegan BF, O’Ceidigh PO, Boaden PJSE (eds.) Biology of benthic organisms. Pergamon Press. Oxford. 1977; pp. 173-183
4. Dixon GB, Zigler KS. Cave-obligate Biodiversity on the Campus of Sewanee: The University of the South, Franklin County, Tennessee. Southeastern Naturalist. 2011; 10: 251-266.
5. Gracia F, Clamor B, Gamundi P, Fornos JJ, Watkinson P. Subaquatic caves of the coastal area of Majorca. Endins. 2011; 35: 103-132.
6. Gracia F, Jaume D. The aquatic fauna in anquihaline and freshwater habitats of Balearic caves. Endins. 2011; 35. 257-268.
7. Idato E, Cinelli F. Fresi E, Mazzella L, Scipione MB. Distribuzione dei popolamenti bentonici di substrato duro lungo un gradient di luce in una grotto marina superficiale dell’isola d’Ischia. Molluschi. Arch. Oceanogr. Limnol. 1983; 20: 113-129.
8. Janssen A, Chevaldonne P, Arbizu PM. Meiobenthic copepod fauna of a marine cave (NW Mediterranean) closely resembles that of deep-sea communities. Mar Ecol Progr Ser. 2013; 479: 99-113. doi 10.3354/meps10207
9. Knight LRFD. The aquatic macro-invertebrate fauna of Swildon's Hole, Mendip Hills, Somerset, UK. Cave and Karst Science. 2011; 38: 81-92.
10. Mazzella L, Cinelli F, Fresi E, Pansini M, Ponticelli MP, Pronzato R. Distribution of benthic phyto- and zoocoenoses along a light gradient in a superficial marine cave: diatoms. Rapp. Comm. Int. expl. Mer Médit. 1979; 25-26: 153-155.
11. Pansini M, Pronzato R, Fresi E, Cinelli F, Mazzella L, Ponticelli MP. Evoluzione delle biocenosi bentoniche di substrato duro lungo un gradient di luce in una grotto marina superficiale. Poriferi. In: Cinelli F, Fresi E, Mazzella L (eds) Atti del 9° Congresso della Società Italiana di Biologia Marina, Lacco Ameno. Olsck ed. Firenze. 1977; pp. 315-330.
12. Sanvicente-Anorve L, Hermoso-Salazar M, Ortigosa J, Solis-Weiss V, Lemus-Santana E. Opistobranch assemblages from a coral reef system: the role of habitat type and food availability. Bull Mar Sci.. 2012; 88: 1061-1074. doi 10.5343/bms.2011.1117
13. Scipione MB, Taramelli E, Fresi E, Cinelli F, Mazzella L. Distribuzione delle biocenosi bentoniche lungo un gradient di luce in una grotto marina superficiale. Anfipodi. Mem Biol Mar Oceanogr. 1981; XI: 1-16.
14. Thomas DJ, Boyd M, Crowell KM, Curtwright AE, Foll MN, Kuehl MM, et al. A Biological Inventory of Meacham Cave (Independence County, Arkansas). J Arkansas Acad Sci.. 2011; 65: 126-132.

**Biogenic**

1. Ashworth EC, Depczynski M, Holmes TH, Wilson SK. Quantitative diet analysis of four mesopredators from a coral reef. J Fish Biol. 2014; 84: 1031-1045. doi 10.1111/jfb.12343
2. Hernaman V, Probert PK, Robbins WD. Trophic ecology of coral reef gobies: interspecific, ontogenetic, and seasonal comparison of diet and feeding intensity. Mar Biol.. 2009; 156: 317-330. doi 10.1007/s00227-008-1085-x
3. Hernandez I, Aguilar C, Gonzalez Sanson G. Trophic webs of reef fishes in northwestern Cuba. I. Stomach contents. Revista De Biologia Tropical. 2008; 56: 541-555.
4. Joubert CSW, Hanekom PB. A study of feeding in some inshore reef fish of the Natal coast, South-Africa. South African Journal of Zoology. 1980; 15: 262-274.
5. Krajewski JP, Floeter SR. Reef fish community structure of the Fernando de Noronha Archipelago (Equatorial Western Atlantic): the influence of exposure and benthic composition. Environmental Biology of Fishes. 2011; 92: 25-40. doi 10.1007/s10641-011-9813-3
6. Matheson RE, Camp DK, Sogard SM, Bjorgo KA. Changes in seagrass-associated fish and crustacean communities on Florida Bay mud banks: The effects of recent ecosystem changes? Estuaries. 1999; 22: 534-551. doi 10.2307/1353216
7. Naumann MS, Jantzen C, Haas AF, Iglesias-Prieto R, Wild C. Benthic Primary Production Budget of a Caribbean Reef Lagoon (Puerto Morelos, Mexico). Plos One. 2013; 8. doi: 10.1371/journal.pone.0082923
8. Pratchett MS, Hoey AS, Wilson SK, Messmer V, Graham NAJ. Changes in Biodiversity and Functioning of Reef Fish Assemblages following Coral Bleaching and Coral Loss. Diversity. 2011; 3: 424-452.
9. Sanvicente-Anorve L, Hermoso-Salazar M, Ortigosa J, Solis-Weiss V, Lemus-Santana E. Opistobranch assemblages from a coral reef system: the role of habitat type and food availability. Bull Mar Sci. 2012; 88: 1061-1074. doi 10.5343/bms.2011.1117

**Hard Bottoms**

1. Azzarello JJ, Smale DA, Langlois TJ, Hakansson E. Linking habitat characteristics to abundance patterns of canopy-forming macroalgae and sea urchins in southwest Australia. Mar Biol Res. 2014; 10: 682-693. doi 10.1080/17451000.2013.841945
2. Buia MC, Gambi MC. Caratterizzazione ambientale e biocenotica e valorizzazione delle aree marine costiere antistanti Cava dell’isola e Punta Caruso (Forio d’Ischia, Napoli). Data Report Ministero dell’Ambiente. Roma. 2000; 267 pp.
3. Cinelli F. Alghe bentoniche di profondità raccolte alla Punta S. Pancrazio nell’Isola d’Ischia (Golfo di Napoli). Giorn Bot Ital. 1971; 105: 207-236.
4. Lenzi M, Gennaro P, Renzi M, Persia E, Porrello S. Spread of *Alsidium corallinum* C. Ag. in a Tyrrhenian eutrophic lagoon dominated by opportunistic macroalgae. Mar Pollut Bull. 2012; 64: 2699-2707. doi 10.1016/j.marpolbul.2012.10.004
5. Lenzi M, Renzi M, Nesti U, Gennaro P, Persia E, Porrello S. Vegetation cyclic shift in eutrophic lagoon. Assessment of dystrophic risk indices based on standing crop evaluations. Estuar Coast Shelf Sci. 2013; 132: 99-107. doi 10.1016/j.ecss.2011.10.006
6. Lyons DA, Arvanitidis C, Blight AJ, Chatzinikolaou E, Guy-Haim T, Kotta J, et al. Macroalgal blooms alter community structure and primary productivity in marine ecosystems. Global Change Biol. 2014; 20: 2712-2724. doi 10.1111/gcb.12644
7. Sanvicente-Anorve L, Hermoso-Salazar M, Ortigosa J, Solis-Weiss V, Lemus-Santana E. Opistobranch assemblages from a coral reef system: the role of habitat type and food availability. Bull Mar Sci. 2012; 88: 1061-1074. doi 10.5343/bms.2011.1117
8. Scipione MB, Fresi E, Chimenz-Gusso C, Gambi MC, Giangrande A, Colognola R. Zonazione delle comunità bentoniche di substrato duro lungo un gradient idrodinamico. Atti del convegno progetto finalizzato Oceanografia e fondi marini: risorse biologiche ed inquinamento. CNR Roma. 1981; pp. 107-117.
9. Vassallo A, Davila Y, Luviano N, Deneb-Amozurrutia S, Guadalupe Vital X, Andres Conejeros C et al. Inventory of invertebrates from the rocky intertidal shore at Montepio, Veracruz, Mexico. Revista Mexicana De Biodiversidad. 2014; 85: 349-362. doi 10.7550/rmb.42628
10. Veras DRA, Martins IX, Matthews-Cascon H. Mollusks: How are they arranged in the rocky intertidal zone? Iheringia Serie Zoologia. 2013; 103: 97-103.
11. Zou D, Gao K. Temperature response of photosynthetic light- and carbon-use characteristics in the red seaweed Gracilariopsis lemaneiformis (Gracilariales, Rhodophyta). J Phycol. 2014; 50: 366-375. doi 10.1111/jpy.12171

**Macroalgae**

1. Buschmann AH, Pereda SV, Varela DA, Rodriguez-Maulen J, Lopez A, Gonzalez-Carvajal L et al. Ecophysiological plasticity of annual populations of giant kelp (Macrocystis pyrifera) in a seasonally variable coastal environment in the Northern Patagonian Inner Seas of Southern Chile. J Appl Phycol. 2014; 26: 837-847. doi 10.1007/s10811-013-0070-z
2. Chaves LTC, Pereira PHC, Feitosa JLL. Coral reef fish association with macroalgal beds on a tropical reef system in North-eastern Brazil. Mar Freshw Res. 2013; 64: 1101-1111. doi 10.1071/mf13054
3. Cinelli F, Fresi E, Mazzella L, Ponticelli MP. Deep algal vegetation of the western Mediterranean. Giorn Bot Ital. 1979; 113: 173-188.
4. Filbee-Dexter K, Scheibling RE. Sea urchin barrens as alternative stable states of collapsed kelp ecosystems. Mar Ecol Prog Ser. 2014; 495: 1-25. doi 10.3354/meps10573
5. Krumhansl KA, Lauzon-Guay J-S, Scheibling RE. Modeling effects of climate change and phase shifts on detrital production of a kelp bed. Ecology. 2014; 95: 763-774. doi 10.1890/13-0228.1
6. Lenzi M, Gennaro P, Renzi M, Persia E, Porrello S. Spread of *Alsidium corallinum* C. Ag. in a Tyrrhenian eutrophic lagoon dominated by opportunistic macroalgae. Mar Pollut Bull. 2012; 64: 2699-2707. doi 10.1016/j.marpolbul.2012.10.004
7. Lenzi M, Renzi M, Nesti U, Gennaro P, Persia E, Porrello S. Vegetation cyclic shift in eutrophic lagoon. Assessment of dystrophic risk indices based on standing crop evaluations. Estuar Coast Shelf Sci. 2013; 132: 99-107. doi 10.1016/j.ecss.2011.10.006
8. Tait LW, Schiel DR. Primary productivity of intertidal macroalgal assemblages: comparison of laboratory and *in situ* photorespirometry. Mar Ecol Progr Ser. 2010; 416: 115-125. doi 10.3354/meps08781
9. Zou D, Gao K. Temperature response of photosynthetic light- and carbon-use characteristics in the red seaweed *Gracilariopsis lemaneiformis* (Gracilariales, Rhodophyta). J Phycol. 2014; 50: 366-375. doi 10.1111/jpy.12171

**Seagrasses**

1. Barnes RSK, Barnes MKS. Biodiversity differentials between the numerically-dominant macrobenthos of seagrass and adjacent unvegetated sediment in the absence of sandflat bioturbation. Mar Environ Res. 2014; 99: 34-43. doi 10.1016/j.marenvres.2014.05.013
2. Bologna PAX, Papagian R, Regetz S, Dale C. Assessment of turtle grass (*Thalassia testudinum* ex Banks Konig) community structure in a UNESCO Biosphere Reserve. J Exp Mar Biol Ecol. 2008; 365: 148-155. doi 10.1016/j.jembe.2008.08.011
3. Brito MdC, Nunez J, San Martin G. Interstitial syllids (Annelids: Polychaeta) associated to seagrass of *Cymodocea nodosa* from the Canary Islands. Avicennia. 2001; 14: 85-100.
4. Buia MC, Gambi MC, Zupo V. Structure and functioning of Mediterranean seagrass ecosystems. Biol Mar Medit. 2000; 7(2): 167-190.
5. Buia MC. Coralline algae epiphytic on *Posidonia oceanica* leaves. J Phycol 27. 1991; 12-12.
6. Buia MC, Zupo V, Mazzella L. Primary production and growth dynamics in *Posidonia oceanica*. PSZNI Mar Ecol. 1992; 13(19): 2-16.
7. Bussotti S, Guidetti P. Fish communities associated with different seagrass systems in the Mediterranean Sea. Naturalista siciliano. 1999; XXIII (suppl): 245-259.
8. Cancemi GL, Buia MC, Mazzella L. Structure and growth dynamics of *Cymodocea nodosa*. Scientia Marina. 2002; 66(4): 365-373.
9. Cantone G Researches on the coast of Somalia – Polychaetous annelids of Magadiscio, Gesira, Bender-Mtoni and Sar-Uanle. Ital J Zool. 1982; 16: 121-139.
10. Carrasco FD, Carbajal W. The distribution of polychaete feeding guilds in organic enriched sediments of San Vicente Bay, Central Chile. Int Rev Hydrobiol. 1998; 83: 233-249. doi 10.1002/iroh.19980830306
11. de Paula AF, Figueiredo MAD, Creed JC. Structure of the macroalgal community associated with the seagrass *Halodule wrightii* Ascherson in the Abrolhos Marine National Park, Brazil. Bot Mar. 2003; 46: 413-424. doi 10.1515/bot.2003.041
12. Ferdie M, Fourqurean JW. Responses of seagrass communities to fertilization along a gradient of relative availability of nitrogen and phosphorus in a carbonate environment. Limnol Oceanogr. 2004; 49: 2082-2094.
13. Fourqurean JW, Willsie A, Rose CD, Rutten LM. Spatial and temporal pattern in seagrass community composition and productivity in south Florida. Mar Biol. 2001; 138: 341-354. Doi: 10.1007/s002270000448
14. Gambi MC, Lorenti M, Russo GF, Scipione MB, Zupo V. Depth and seasonal distribution of some groups of the vagile fauna of the *Posidonia oceanica* leaf stratum: structural analysis. PSZNI Mar Ecol. 1992; 13(1): 17-39.
15. Gartner A, Tuya F, Lavery PS, McMahon K. Habitat preferences of macroinvertebrate fauna among seagrasses with varying structural forms. J Exp Mar Biol Ecol. 2013; 439: 143-151. doi 10.1016/j.jembe.2012.11.009
16. Greenway M. Trophic relationships of macrofauna within a Jamaican seagrass meadow and the role of the echinoid *Lytechinus variegatus* (Lamark). Bull Mar Sci.1995; 56: 719-736.
17. Hall MO, Durako MJ, Fourqurean JW, Zieman JC. Decadal changes in seagrass distribution and abundance in Florida Bay. Estuaries. 1999; 22: 445-459. doi 10.2307/1353210
18. Hansen JP, Sagerman J, Wikstrom SA. Effects of plant morphology on small-scale distribution of invertebrates. Mar Biol. 2010; 157: 2143-2155. doi 10.1007/s00227-010-1479-4
19. Harmelin-Vivien MI, Francour P. Trawling or visual census? Methodological bias in the assessment of fish populations in seagrass beds. PSZNI Mar Ecol. 1992; 13(1): 41-51.
20. Jeong SJ, Yu OH, Suh HL. Secondary production of *Monocorophium acherusicum* (Amphipoda, Corophiidae) in a seagrass bed (*Zostera marina*). J Korean Fisher Soc. 2006; 39: 236-241.
21. Kutser T, Vahtmaee E, Roelfsema CM, Metsamaa L. Photo-library method for mapping seagrass biomass. Estuar Coast Shelf Sci. 2007; 75: 559-563. doi 10.1016/j.ecss.2007.05.043
22. Lanera P. Studio sulla fauna associate a prati della fanerogama marina *Cymodocea nodosa* (Ucria) Ashers. dell’isola d’Ischia. Degree thesis. Zoology department. University Federico II of Naples. 1987; 119 pp.
23. Lavery PS, Reid T, Hyndes GA, Van Elven BR. Effect of leaf movement on epiphytic algal biomass of seagrass leaves. Mar Ecol Progr Ser. 2007; 338: 97-106. doi 10.3354/meps338097
24. Masese FO, Kitaka N, Kipkemboi J, Gettel GM, Irvine K, McClain ME. Macroinvertebrate functional feeding groups in Kenyan highland streams: evidence for a diverse shredder guild. Freshw Sci. 2014; 33: 435-450 doi 10.1086/675681
25. Matheson RE, Camp DK, Sogard SM, Bjorgo KA. Changes in seagrass-associated fish and crustacean communities on Florida Bay mud banks: The effects of recent ecosystem changes? Estuaries. 1999; 22: 534-551. doi 10.2307/1353216
26. Mazzella L, Buia MC, Gambi MC, Lorenti M, Russo GF, Scipione MB, Zupo V. A review on the trophic organization in the *Posidonia oceanica* ecosystem. In: Cinelli F, Fresi E, Lorenzi C, Mucedola A (editors.) La Posidonia oceanica. Rivista marittima. 1995; 12: 40-47.
27. Mazzella L, Scipione MB, Gambi MC, Buia MC, Lorenti M, Zupo V, Cancemi G. The Mediterranean seagrasses *Posidonia oceanica* and *Cymodocea nodosa*. A comparative overview. The first International Conference on the Mediterranean Coastal Environment. MEDCOAST 93, Antalya, Turkey 1. 1993; pp. 103-116.
28. Plus M, Deslous-Paoli JM, Auby I, Dagault F. Factors influencing primary production of seagrass beds (*Zostera noltii* Hornem.) in the Thau lagoon (French Mediterranean coast). J Exp Mar Biol Ecol. 2001; 259: 63-84. doi 10.1016/s0022-0981(01)00223-4
29. Potter IC, Chalmer PN, Tiivel DJ, Steckis RA, Platell ME, Lenanton RCJ. The fish fauna and finfish fishery of the Leschenault Estuary in south-western Australia. J Royal Soc West Austral. 2000; 83: 481-501.
30. Scipione MB. Do studies on functional groups give more insight to amphipod biodiversity? Crustaceana. 2013; 86: 955-1006. doi 10.1163/15685403-00003209
31. Velimirov B. Organic matter derived from seagrass meadows: origin, properties and quality of particles. PSZNI Mar Ecol. 1987; 8: 143-173.
32. Vizzini S. Analysis of the trophic role of Mediterranean seagrasses in marine coastal ecosystems: a review. Botanica Marina. 2009; 52(5): 383-393.
33. Zupo V. The use eof feeding indices for the study of food webs: an application to a *Posidonia oceanica* ecosystem. Coenoses. 1993; 8: 85-95.

**Soft bottoms**

1. Barnes RSK, Barnes MKS. Biodiversity differentials between the numerically-dominant macrobenthos of seagrass and adjacent unvegetated sediment in the absence of sandflat bioturbation. Mar Environ Res.. 2014; 99: 34-43. doi 10.1016/j.marenvres.2014.05.013
2. Gambi MC. Studio di Fattibilità per l’istituzione dell’area marina protetta “Regno di Nettuno” (Isole di Ischia, Procida e Vivara) Seconda fase. Ministero dell’Ambiente. Roma. 2001; 345 pp.
3. Gimenez L, Venturini N, Kandratavicius N, Hutton M, Lanfranconi A, Rodriguez M et al. Macrofaunal patterns and animal-sediment relationships in Uruguayan, estuaries and coastal lagoons (Atlantic coast of South America). J Sea Res. 2014; 87: 46-55. doi 10.1016/j.seares2013.12.005
4. Levinton, JS. Marine Ecology. Prentice Hall Inc., Englewood Cliffs, New Jersey. 1982; 526 pp.
5. Naumann MS, Jantzen C, Haas AF, Iglesias-Prieto R, Wild C. Benthic Primary Production Budget of a Caribbean Reef Lagoon (Puerto Morelos, Mexico). Plos One. 2013; 8: doi 10.1371/journal.pone.0082923
6. Veiga P, Rubal M, Cacabelos E, Maldonado C, Sousa-Pinto I. Spatial variability of macrobenthic zonation on exposed sandy beaches. J Sea Res.. 2014; 90: 1-9. doi 10.1016/j.seares2014.02.009
7. Wong MC, Dowd M. Role of Invasive Green Crabs in the Food Web of an Intertidal Sand Flat Determined from Field Observations and a Dynamic Simulation Model. Estuaries and Coasts. 2014; 37:1004-1016 doi 10.1007/s12237-013-9728-7

**Harbours**

1. Carrasco FD, Carbajal W. The distribution of polychaete feeding guilds in organic enriched sediments of San Vicente Bay, Central Chile. International Rev Hydrobiol. 1998; 83: 233-249. doi 10.1002/iroh.19980830306
2. Chimenz C, Fresi E, Brunetti R. Ricerche sui popolamenti bentonici di substrato duro del porto d’Ischia. Ascidiacei. Cah Biol Mar. 1985; XXVI: 15-33.
3. Chimenz C, Fresi E, Cinelli F, Mazzella L, Di Costanzo S. Ricerche sui popolamenti bentonici di substrato duro del porto d’Ischia. Briozoi. Mem. Biol. Mar. Oceanogr. 1981a; XI: 187-206.
4. Chimenz C, Fresi E, Pepe R, Cinelli F, Mazzella L, Scipione MB. Ricerche sui popolamenti bentonici di substrato duro del porto d’Ischia. Cirripedi opercolati. Quaderni Lab. Tecnol. Tesca Ancona. 1981b; 3: 493-504.
5. Cinelli F, Fresi E, Mazzella L. Ricerche sui popolamenti bentonici di substrato duro del porto d’Ischia. I. Infralitorale fotofilo (macrofite e isopodi liberi). Arch- Oceanogr. Limnol. 1976; 18: 169-188.
6. Fresi E, Colognola R, Gambi MC, Giangrande A, Scardi M. Ricerche sui popolamenti bentonici di substrato duro del Porto d’Ischia. Infralitorale fotofilo. Policheti (I). Cah. Biol. Mar. 1983; 24: 1-19.
7. Fresi E, Colognola R, Gambi MC, Giangrande A, Scardi M. Ricerche sui popolamenti bentonici di substrato duro del Porto d’Ischia. Infralitorale fotofilo. Policheti (II) Cah. Biol. Mar. 1984; 25: 33-47.
8. Fresi E., Chimenz C, Pepe R. Ricerche sui popolamenti bentonici di substrato duro del porto d’Ischia. Cirripedi opercolati: analisi statistic-matematica. Mem. Biol. Mar. Oceanogr. 1981; XI: 207-217.
9. Fresi E, Maggiore F. Ricerche sui popolamenti bentonici di substrato duro del Porto d’Ischia: isopodi. Mem. Biol. Mar. Oceanogr. 1984; XVI: 17-29.
10. Greenway M. Trophic relationships of macrofauna within a Jamaican seagrass meadow and the role of echinoid Lythechinus variegatus (Lamark). Bul Mar Sci. 1995; 56: 719-736.
11. Hansen JP, Sagerman J, Wikstrom SA. Effects of plant morphology on small-scale distribution of invertebrates. Mar Biol. 2010; 157: 2143-2155. doi 10.1007/s00227-010-1479-4
12. Hereu B, Zabala M, Sala E. Multiple controls of community structure and dynamics in a sublittoral marine environment. Ecology. 2008; 89: 3423-3435. doi 10.1890/07-0613.1
13. Hossain M, Arhonditsis GB, Koops MA, Minns CK. Towards the development of an ecosystem model for the Hamilton Harbour, Ontario, Canada. J Great Lakes Res. 2012; 38: 628-642. doi 10.1016/j.jglr.2012.09.015
14. Idato E, Chimenz C, Cinelli F, Fresi E, Mazzella L, Scipione MB. Ricerche sui popolamenti bentonici di substrato duro del porto d’Ischia. IV. Molluschi. Seconda nota. Atti Accademia dei Fisiocratici, Siena. 1980; 2: 201-213.
15. Idato E, Cinelli F, Fresi E, Mazzella L, Ponticelli MP, Scipione MB. Ricerche sui popolamenti bentonici di substrato duro del porto d’Ischia.Infralitorale fotofilo: IV Molluschi. Quaderni Lab. Tecnol. Pesca Ancona. 1981; 3: 519-534.
16. Masese FO, Kitaka N, Kipkemboi J, Gettel GM, Irvine K, McClain ME. Macroinvertebrate functional feeding groups in Kenyan highland streams: evidence for a diverse shredder guild. Freshw Sci. 2014; 33: 435-450. doi 10.1086/675681
17. Mazzella L, Cinelli F, Fresi E, Ponticelli MP. Ricerche sui popolamenti bentonici di substrato duro del porto d’Ischia. Infralitorale fotofilo: II. Microflora a diatomee. Giorn Bot Ital. 1978; 112: 13-27.
18. Metcalfe KN, Glasby CJ. Diversity of polychaeta (Annelida) and other worm taxa in mangrove habitats of Darwin Harbour, northern Australia. J Sea Res.. 2008; 59: 70-82. doi 10.1016/j.seares.2007.06.002
19. Scipione MB, Carnevale G, Cinelli F, Fresi E, Mazzella L, Ponticelli MP, Taramelli E. Ricerche sui popolamenti bentonici di substrato duro del porto d’Ischia. Infralitorale fotofilo: III Anfipodi. Quaderni Lab. Tecnol. Pesca Ancona. 1981; 3: 505-518.
20. Sheridan P. Benthos of adjacent mangrove, seagrass and non-vegetated habitats in Rookery Bay, Florida, USA. Estuar Coast Shelf Sci. 1997; 44: 455-469. doi 10.1006/ecss.1996.0125
21. Terlizzi A, Conte E, Zupo V, Mazzella L. Biological succession on silicone fouling-release surfaces: Long-term exposure tests in the harbour of Ischia, Italy. Biofouling. 2000; 15: 327-342.

**Biotope typologies**

1. Barnes M, Gibson RN. Trophic relationships in the marine environment. Proceedings of the 24^th^ European Marine Biology Symposium. Aberdeen University press. UK. 1990; 642 pp.
2. Ferdie M, Fourqurean JW. Responses of seagrass communities to fertilization along a gradient of relative availability of nitrogen and phosphorus in a carbonate environment. Limnol Oceanogr. 2004; 49: 2082-2094.
3. Fourqurean JW, Willsie A, Rose CD, Rutten LM. Spatial and temporal pattern in seagrass community composition and productivity in south Florida. Mar Biol. 2001; 138: 341-354. doi 10.1007/s002270000448
4. Freed S, Granek EF. Effects of Human Activities on the World's Most Vulnerable Coral Reefs: Comoros Case Study. Coastal Managemen. 2014; 42: 280-296. doi 10.1080/08920753.2014.904261
5. Hall MO, Durako MJ, Fourqurean JW, Zieman JC. Decadal changes in seagrass distribution and abundance in Florida Bay. Estuaries. 1999; 22: 445-459. doi 10.2307/1353210
6. Kaldy JE. Effect of temperature and nutrient manipulations on eelgrass *Zostera marina* L. from the Pacific Northwest, USA. J Exp Mar Biol Ecol. 2014; 453: 108-115. doi 10.1016/j.jembe.2013.12.020
7. Kraemer GP, Mazzella L. Nitrogen assimilation and growth dynamics of the Mediterranean seagrasses *Posidonia oceanica*, *Cymodocea nodosa* and *Zostera noltii*. In: Kuo J., Phillips RC, Walker D, Kirkman H (editors) Seagrass biology: proceedings of an International workshop. Rottnest Island, Western Australia. 1996; pp. 181-190.
8. Lapointe BE, Barile PJ, Yentsch CS, Littler MM, Littler DS, Kakuk B. The relative importance of nutrient enrichment and herbivory on macroalgal communities near Norman's Pond Cay, Exumas Cays, Bahamas: a "natural" enrichment experiment. J Exp Mar Biol Ecol. 2004; 298: 275-301. doi 10.1016/s0022-0981(03)00363-0
9. Lorenti M, Mazzella L, Buia MC. Light limitation of *Posidonia oceanica* (L) Delile growth at different depths. Rapp Comm Int expl Mer Médit. 1995; 34: 34-34.
10. McClanahan TR. Recovery of functional groups and trophic relationships in tropical fisheries closures. Mar Ecol Progr Ser. 2014; 497: 13-23. doi 10.3354/meps10605
11. Plus M, Deslous-Paoli JM, Auby I, Dagault F. Factors influencing primary production of seagrass beds (*Zostera noltii* Hornem.) in the Thau lagoon (French Mediterranean coast). J Exp Mar Biol Ecol. 2001; 259: 63-84. doi 10.1016/s0022-0981(01)00223-4
12. Pratt DR, Lohrer AM, Pilditch CA, Thrush SF. Changes in Ecosystem Function Across Sedimentary Gradients in Estuaries. Ecosystems. 2014; 17: 182-194. doi 10.1007/s10021-013-9716-6
13. Vasapollo, C, Gambi, MC. Spatio-temporal variability in *Posidonia oceanica* seagrass meadows of the Western Mediterranean: shoot density and plant features. Aquat Biol. 2012; 16 (2): 163-175. doi: 10.3354/ab00456

**General literature for RAFI table calculations**

1. Zupo V. 1993 The use of feeding indices for the study of food webs: an application on Posidonia oceanica ecosystem. Coenoses 8(2), 85-95.
2. Jørgensen SE. 1979 Handbook of environmental data and ecological parameters. Pergamon press. Oxford, 1162 pp.
3. Coll M, Libralato S. 2012 Contributions of food web modelling to the ecosystem approach to marine resource management in the Mediterranean Sea. Fish and Fisheries 13, 60–88.
4. Novak M. 2013 Trophic omnivory across a productivity gradient: intraguild predation theory and the structure and strength of species interactions. Proc. Roy. Soc-B 280, 1766. (doi: 10.1098/rspb.2013.1415)
5. Gambi MC, Lorenti M, Russo GF, Scipione MB, Zupo V. 1992 Depth and seasonal distribution of some groups of the vagile fauna of the Posidonia oceanica leaf stratum- structural and trophic analyses. PSZNI Mar. Ecol. 13(1), 17-39. (doi: 10.1111/j.1439-0485.1992.tb00337.x)
6. Lorenti M, Scipione B. 1990 Relationships between trophic structure and diel migrations of isopods and amphipods in a Posidonia oceanica bed off the island of Ischia (Gulf of Naples, Italy). Rapp. Proc. R. Comm. Int. Exp. Sci. Mer Médit. 32, 17-17.
7. Liu H, Fogarty MJ, Hare JA, Hsieh CH, Glaser SM, Ye H et al. 2014 Modelling dynamic interactions and coherence between marine zooplankton and fishes linked to environmental variability. J. Mar. Sys. 131, 120-129. (doi: 10.1016/j.jmarsys.2013.12.003)
8. Buia MC, Zupo V, Mazzella L. 1992 Primary production and growth dynamics in Posidonia oceanica. PSZNI Mar. Ecol. 13(19), 2-16.
9. Mazzella L, Zupo V. 1995 Reti trofiche e flussi di energia nei sistemi a fanerogame marine. Giorn. Bot. Ital. 129 (1), 337-350.
10. Fransz HG, Mommaerts JP, Radach G. 1991 Ecological modelling of the North sea. Netherlands J. Sea Res. 28(1-2), 67-140. (doi: 10.1016/0077-7579(91)90005-L)
11. Barnes M, Gibson RN. 1990 Trophic relationships in the marine environment. Proc. 24th European Mar. Biol. Symp. Aberdeen University press. UK., 642 pp.
12. Bizina EV. 2000 Predators, resources and trophic cascades in the regulation of plankton communities in freshwater oligotrophic lakes. Zhurnal obshchei biologii. 61(6), 601-615.
13. Morris DJ, Speirs DC, Cameron AI, Heath MR. 2014 Global sensitivity analysis of an end-to-end marine ecosystem model of the North Sea: Factors affecting the biomass of fish and benthos. Ecological modelling. 273, 251-263. (doi: 10.1016/j.ecolmodel.2013.11.019)

**Supporting Literature for Table 4 in the main text**

1. Terlizzi A, Russo GF. 1988 The molluscan taxocoene of differently-exposed Cymodocea nodosa beds: year-long structural patterns and sampling methods. Boll. Malacol. 33 (5-8), 77-82.
2. Lanera P, Gambi MC. 1993 Polychaete distribution in some Cymodocea nodosa meadows around the island of Ischia (Gulf of Naples Italy). Oebalia 19, 89-103.
3. Cigliano M, Gambi MC. 2007 The long hot summer: a firther mortality event of gorgonians along the phlegrean islands (Tyrrhenian sea) Biol. Mar. Medit. 14 (2), 292-293.
4. Cinelli F. 1971 Alghe bentoniche di profondità raccolte alla punta S. Pancrazio nell'isola di Ischia (Golfo di Napoli). Plant Biosystems. 105(5), 207-236. (doi: 10.1080/11263507109426526)
5. Gambi MC, Cigliano M, Iacono B. 2006 Record of a mass mortality event of gorgonarians on the coast of Ischia and Procida Islands (Gulf of Naples, Tyrrhenian Sea). Biol. Mar. Medit. 13 (1), 583-587.
6. Scipione MB, Taramelli E, Fresi E, Cinelli F, Mazzella L. 1981 Distribuzione delle biocenosi bentoniche lungo un gradiente di luce in una grotta marina superficiale: anfipodi. Mem. Biol. Mar. Oceanogr. 11 (1), 1-16.
7. Pansini M, Pronzato R, Fresi E, Cinelli F, Mazzella L, Ponticelli MP. 1977 Evoluzione delle biocenosi bentoniche di substrato duro lungo un gradiente di luce in una grotta marina superficiale: poriferi. Atti Congr. Soc. Ital. Biol. Mar. 9, 315-330.
8. Dappiano M, Gambi MC. 2004 New data on occurrence of thermophile Scleractinia (Cnidaria, Anthozoa) in the Phlaegrean Islands (Ischia, Procida, Vivara, Gulf of Naples), with special attention to Astroides calycularis. Biogeographia 25, 31-46.
9. Gambi MC, Dappiano M, Lorenti M, Iacono B, Flagella S, Buia MC. 2005 “Chronicle of a death foretold”. Features of a Posidonia oceanica bed impacted by sand extraction in the island of Ischia (Gulf of Naples, Italy). In: Ozhan E. (editors), Proceedings of the Seventh International Conference on the Mediterranean Coastal Environment, MED-COAST 05, Kusadasi, Turkei, pp. 441-450.
10. Flagella S, Borriello I, Gambi MC, Buia MC. 2006 Responses of Posidonia oceanica to environmental disturbances. Biol. Mar. Medit. 13(4), 215-219.
11. Chimenz C, Fresi E, Brunetti R. 1985 Ricerche sui popolamenti bentonici di substrato duro del porto d’Ischia. Ascidiacei. Cah. Biol. Mar. XXVI, 15-33.
12. Cinelli F, Fresi E, Mazzella L. 1976 Ricerche sui popolamenti bentonici di substrato duro del porto d’Ischia. I. Infralitorale fotofilo (macrofite e isopodi liberi). Arch. Oceanogr. Limnol. 18, 169-188.
13. Buia MC, Gambi MC. 2000 Caratterizzazione ambientale e biocenotica e valorizzazione delle aree marine costiere antistanti Cava dell’isola e Punta Caruso (Forio d’Ischia, Napoli). Data Report Ministero Ambiente. Roma, 75 pp.
14. Garrard SL, Gambi MC, Scipione MB, Patti FP, Lorenti M, Zupo V et al. 2014 Indirect effects may buffer negative responses of seagrass invertebrate communities to ocean acidification. J. Exp. Mar. Biol. Ecol. 461, 31–38.
15. Arnold T, Mealey C, Leahey H, Miller AW, Hall-Spencer JM, Milazzo M, Maers K. 2012 Ocean Acidification and the Loss of Phenolic Substances in Marine Plants. Plos One 7(4), e35107. (doi: 10.1371/journal.pone.0035107)
16. Kroeker KJ, Gambi MC, Micheli F. 2013 Community dynamics and ecosystem simplification in a high-CO_2_ ocean. Proc. Nat. Acad. Sci. USA 110 (31), 12721-12726. (doi: 10.1073/pnas.1216464110)
17. Pittman SJ, Hile SD, Jeffrey CFG, Caldow C, Kendall MS, Monaco ME, Hillis-Starr Z. 2008 Fish assemblages and benthic habitats of Buck Island Reef National Monument (St. Croix, U.S. Virgin Islands) and the surrounding seascape: a characterization of spatial and temporal patterns. NOAA Technical Memorandum NOS NCCOS 71, 1-80.
18. Schiel DR, Andrew NL, Foster MS. 1995 The structure of subtidal algal and invertebrate assemblages at the Chatham islands, New Zealand. Mar. Biol. 123(2), 355-367. (doi: 10.1007/BF00353627)
19. Alvarado JJ, Chiriboga A. 2008 Distribution and abundance of shallow water echinoderms from Cocos Island, Costa Rica, Eastern Pacific. Rev. Biologia Tropical. 56, 99-111.
